# Glycolytic ATP production enables rapid mammalian cell growth

**DOI:** 10.1101/2025.10.30.685682

**Published:** 2025-10-31

**Authors:** Matthew A. Kukurugya, Shuying Zhang, Brandon T. Ha, Alex E. Ekvik, Denis V. Titov

**Affiliations:** 1 Department of Molecular & Cell Biology, University of California; Berkeley CA, 94720; 2 Department of Nutritional Sciences & Toxicology, University of California; Berkeley CA, 94720; 3 Center for Computational Biology, University of California; Berkeley CA, 94720

## Abstract

Proliferating cells must produce ATP rapidly enough to meet the energy demands of growth and maintenance. While microbes show a linear coupling between ATP production rate and growth, whether this principle holds in mammalian cells has remained unclear and it has been suggested that most ATP is allocated to cell maintenance, regardless of growth rate. Here, we quantified lactate production, oxygen consumption, and proliferation across twelve mammalian cell lines and found a strong linear relationship between total ATP production and growth with the majority of ATP allocated to macromolecular synthesis. By inhibiting glycolysis, inhibiting respiration, or reducing translation, cells shift along this ATP–growth line in predictable directions, indicating bidirectional coupling between ATP supply and demand. A genetically encoded ATP hydrolysis sink increased ATP turnover yet slowed proliferation, demonstrating that ATP production capacity can limit growth. Together, these results show that respiration alone cannot generate enough ATP to support the growth rates of rapidly dividing cells, whereas glycolysis can. Our results provide a quantitative rationale for the Warburg Effect, where cells rely on glycolysis to achieve doubling times faster than 30 hours. Our results establish ATP production rate as a quantitative constraint on growth across species.

Adenosine triphosphate (ATP) serves as the universal energy currency of the cell, mediating the coupling of catabolic and anabolic reactions essential for cellular function. Proliferating cells require ATP to synthesize the biomass necessary for division and the production of two daughter cells. ATP production and cell growth have been extensively studied in microbes^1–6^, where a linear relationship has been established between ATP production and growth rate^7,8^. Maintenance ATP production, required for non-growth activities such as ion gradient and cell motility, accounts for a small fraction of the total ATP expenditure in microbial cells near their maximal growth rate. The relationship between ATP production and growth rate has not been systematically investigated in mammalian cells. Only one study exists and has suggested that the majority of ATP produced in mammalian cells is for cellular maintenance and ATP production only modestly increases as a function of growth rate.^9,10^ Furthermore, ATP production rates measurements from solid tumors in mice have suggested to be lower than those in native, nonproliferating tissues; however, metastasizing tumors appear to exhibit higher ATP production rates than their tumors of origin^11^. Given the discrepancies between well-defined microbial bioenergetic principles and less characterized energy dynamics in mammalian cells, a quantitative relationship between ATP production and cellular growth rate in mammalian cells remains to be established.

Rapidly proliferating cells use aerobic glycolysis or fermentation to generate a large fraction of their ATP, known as the Warburg Effect^12,13^. Conversely, slow-growing or differentiated cells use respiration for ATP production. In microbes, the glycolytic rate increases with growth for a given nutrient limitation^14^. A previous study concluded that the glycolytic rate did not correlate with growth rate in mammalian cells; however, this interpretation was influenced by the use of cell count, whereas correcting for biomass as the normalization metric correlates glycolytic activity with growth^15^. Our previous work demonstrates that cells utilize glycolysis to maximize their ATP production rate when ATP demand is high and glucose is abundant^14^. Our conclusion rests on two assumptions. First, glycolysis has a higher proteome efficacy than respiration, which has been shown across organisms that exhibit the Warburg Effect^14,16,17,8^. Second, the ATP production must be limiting for growth. The latter has yet to be experimentally validated in mammalian cells.

Typically, cancer cells possess a spare capacity for both respiration and glycolysis, allowing compensation when one pathway is inhibited^18,19^. Furthermore, some evidence indicates that cancer cells may have excess ATP^20,21^ and increased ATP consumption could potentially drive faster proliferation^20,22^. Yet, in *E. coli*, elevated ATP demand reduces growth rate, despite the upregulation of glycolysis to increase ATP synthesis^23^. Similar to *E. coli*, elevated ATP demand in mammalian cells invokes increased ATP production through glycolysis; however, its effect on growth rate remains unclear^24^. Therefore, the limit of ATP demand on growth and the extent that glycolysis is necessary has yet to be quantified in mammalian cells.

Here, we rigorously quantify the ATP demands of mammalian cell growth and demonstrate that glycolysis is necessary to achieve doubling times faster than ~24 hours. We report an extensive dataset of over 7500 individual measurements of glycolytic rates, respiratory rates, cell sizes, and growth rates in a dozen mammalian cell lines in the presence of perturbations of ATP production and demand. Our data demonstrates a strong linear relationship between ATP production rate and growth across mammalian cells, which is in agreement with published data for *E. coli* and *S. cerevisiae*. Using orthogonal perturbations of ATP production and cell growth, we demonstrate a causal relationship between ATP production and growth rate. Contrary to previous reports^9,10,25^, we show that almost all of ATP production in mammalian cells goes into fueling cell growth at the 24-hour doubling time, suggesting that, similar to *E. coli* and *S. cerevisiae*, growth is the major energy-consuming process in proliferating mammalian cells. In addition, our predictions show that respiration alone cannot sustain the ATP demands observed in fastest-growing cells of all three species, providing additional evidence for the hypothesis that the benefit of the Warburg Effect is to allow faster ATP production in rapidly growing cells.

## Results

### ATP production rate increases linearly with growth rate

**Extended Data Fig. 1:**
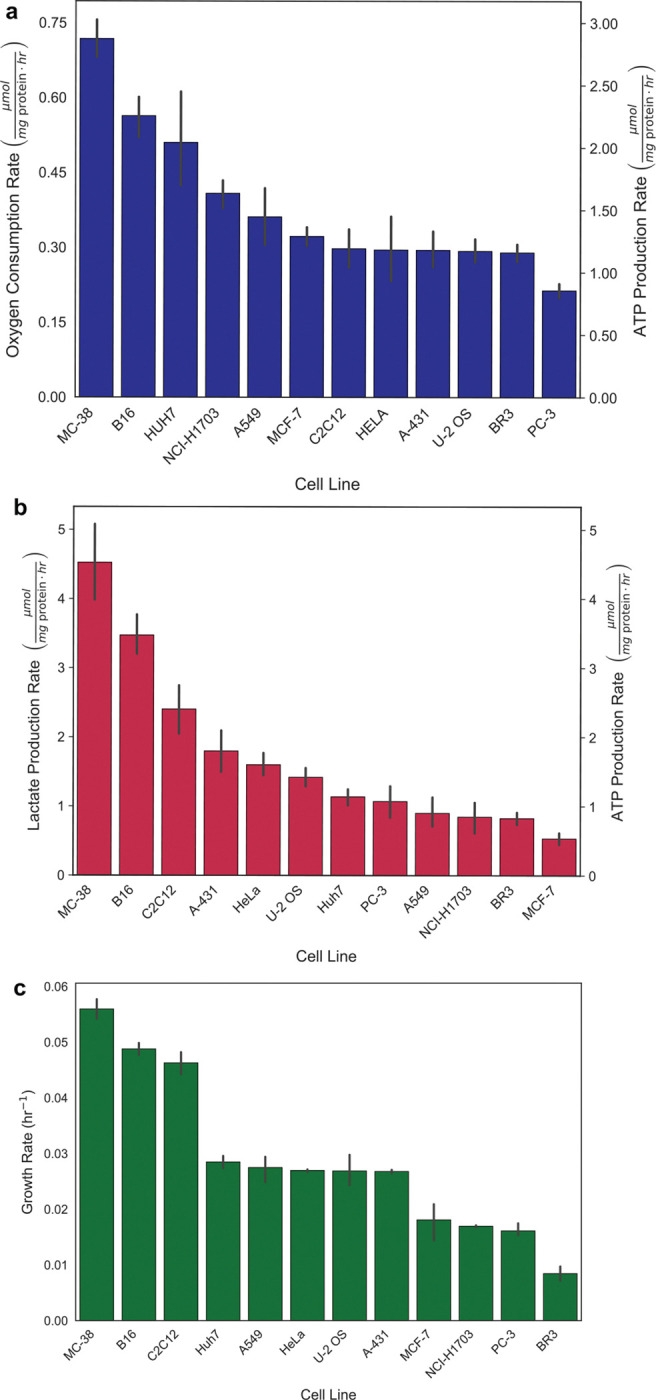
Oxygen consumption rate, lactate production rate, and growth rate of twelve mammalian cells lines. a, Oxygen consumption rates (μmol mg protein^−1^ hr^−1^), b, lactate production rates (μmol mg protein^−1^ hr^−1^) and c, growth rates (hr^−1^) of twelve mammalian cell lines. The error bars in represent the 95% confidence interval.

**Extended Data Fig. 2:**
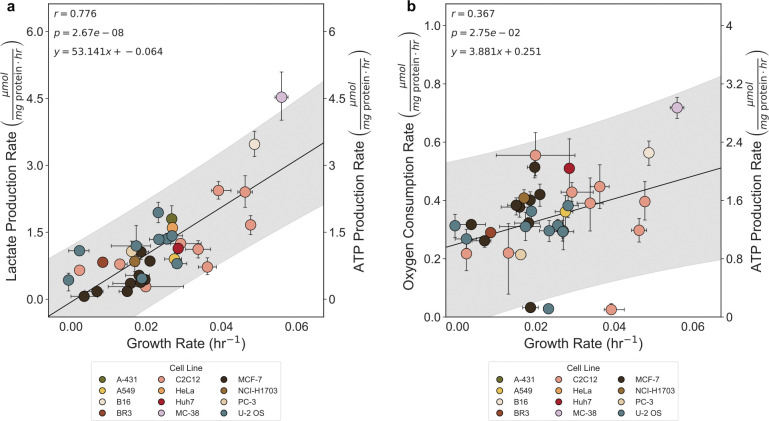
Correlation of lactate production rate and oxygen consumption rate with growth rate in mammalian cells. a, Lactate production rate (μmol mg protein^−1^ hr^−1^) b, Oxygen consumption rate (μmol mg protein^−1^ hr^−1^) mammalian cell culture lines. The error bars in represent the 95% confidence interval calculated from 10,000 bootstrap iterations.

**Fig 1:**
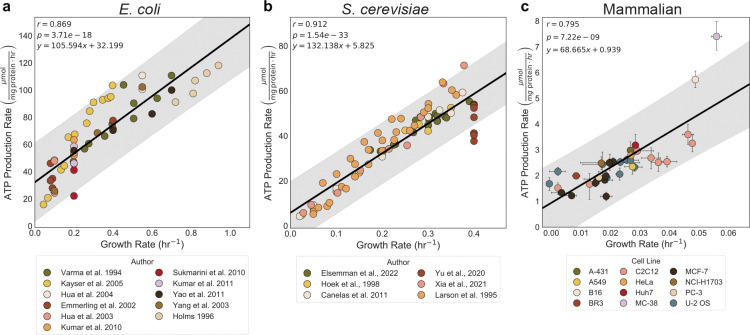
ATP production rate and growth rate are highly correlated across *E. coli*, *S. cerevisiae*, and mammalian cells. a-c, The correlation between ATP production rate (μmol mg protein^−1^ hr^−1^) and growth rate (hr^−1^) in *E. coli* (a), *S. cerevisiae* (b), and twelve mammalian cell lines (c). Point colors represent data sources or cell lines. Note that all mammalian cell data was generated in the current study. The shaded band shows the 95% prediction interval, the range in which 95% of future individual observations are expected to fall around the fitted regression line. The error bars in represent the 95% confidence interval calculated from 10,000 bootstrap iterations.

In proliferating cells, the ATP production rate must increase with increasing growth rate as it supports biosynthesis. Increasing ATP production rates with faster growth have been well-appreciated in microbes^8,26–28^. We were able to corroborate these findings by demonstrating a linear increase in the ATP production rate (μmol mg protein^−1^ hr^−1^) with growth rate through analyzing curated data from *E. coli*^28–38^ (r = 0.869, p = 3.71e-18) and *S. cerevisiae*^8,27,39–42^ (r = 0.912, p = 1.54e-33) (Fig. 1 a and b). However, only one study has investigated the relationship between ATP production rate and cell growth in a single mammalian cell type, highlighting the need for more comprehensive studies^9^. We tested the correlation between ATP production rate and proliferation rate in twelve mammalian cell culture lines with varied chemical perturbations and media compositions by measuring lactate production, oxygen consumption, and growth rates of mammalian cell lines (Extended Data Fig 1). We selected mammalian cells with varying sizes, growth rates, and tissue of origin to demonstrate that this is a conserved relationship across cell types. Each data point represents a composite of over 200 individual measurements, derived from three experimental modalities: (1) a nuclei-staining plate-based growth assay, (2) a colorimetric lactate assay, and (3) a Seahorse assay. Each modality was performed independently repeated three times. Using the summed ATP production rate from oxygen consumption and lactate production, we found a strong correlation between ATP production rate and faster growth for mammalian cells (R^2^ = 0.801, p = 8.43e-5) (Fig. 1c). The strong correlation between lactate production and growth rate (r = 0.776, p = 2.67e-8) supports the hypothesis that glycolysis plays a critical role in meeting the elevated ATP demands of rapidly proliferating cells (Extended Data Fig 2a). We found that oxygen consumption had a weak positive correlation with growth rate (r = 0.367, p = 2.75e-2) (Extended Data Fig 2b). Our findings underscore the conserved relationship between ATP production rate and growth across diverse cell types, including mammalian cells.

**Fig. 2:**
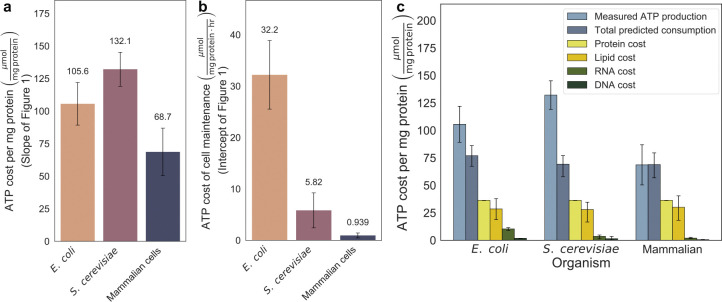
The majority of ATP produce in fast-growing cells supports protein synthesis, not maintenance. a, Slope of the linear fit between ATP production rate (μmol mg protein^−1^ hr^−1^) and growth rate (hr^−1^) for *E. coli*, *S. cerevisiae*, and mammalian cells, which represents the ATP cost of making 1 mg of cell protein equivalent. The error bars in represent the 95% confidence interval of the slope. b, Y-intercepts for the ATP production vs, growth rate, representing the ATP production required to sustain maintenance in non-dividing cells. The error bars in represent the 95% confidence interval. c, Comparison between the experimentally measured ATP cost of growth (slopes from panel Fig. 2a) and the theoretical biosynthetic cost of synthesizing proteins, lipids, RNA and DNA, based on known ATP requirements for macromolecular biosynthesis. The error bars in represent the 95% confidence interval.

When comparing the ATP production rates required to support growth across the three organisms, we observed similar slopes: 105 μmol mg protein^−1^ for *E. coli*, 132 μmol mg protein^−1^ for *S. cerevisiae*, and 68 μmol mg protein^−1^ for mammalian cells. (Fig. 2a). Our results suggest a conserved energetic investment in ATP per unit of biomass synthesized across organisms. The lower apparent ATP cost in mammalian cells may reflect culture in nutrient-replete media, where preformed amino acids, lipids, and nucleotides reduce biosynthetic demand, in contrast to microbes grown in minimal media that must synthesize most biomass precursors *de novo*. Each regression crossed the y-axis above zero, consistent with a growth-independent maintenance ATP requirement (Fig. 2b). Maintenance energy supports essential processes such as ion homeostasis, cell motility, protein turnover, pH regulation, and DNA repair^2^. Unlike the ATP required for growth, we observed a log-fold decrease in the required ATP production rate from the maintenance of a mg of protein from *E. coli* to mammalian cells (Fig 2b). A previous study observed a similar magnitude of change in the maintenance energy requirements of mouse LS cells compared to those of the fungus *P. chrysogenum* and the bacterium *A. aerogenes*^9^. We speculate that the observed differences in maintenance energy may be associated with changes in the surface-area-to-volume ratio, which scale similarly across organisms and align with the principles described by Kleiber’s law^43,44^. Importantly, we find that required ATP maintenance represents only a minor fraction of the total ATP consumption in rapidly growing cells. While some have proposed that ATP demand for proliferation may be less than for basal maintenance^10,45,46^, our data indicate that ATP production must scale substantially with growth rate.

To further corroborate our findings, we compared the observed slopes to the known biochemical costs associated with synthesizing proteins, lipids, and nucleotides in proportions consistent with those found in each organism (Fig. 2c). The theoretical component was estimated by summing the ATP costs associated with the biosynthesis of proteins, lipids, and nucleotides, weighted by their experimentally measured mass fractions within each organism’s biomass composition. From our analysis, we found that our empirical ATP production rates only slightly exceeded the theoretical biosynthetic cost (Fig 2c). This theoretical demand accounted for ~85% of the total empirical ATP production for *E. coli*, ~65% for *S. cerevisiae*, and 100% for mammalian cells, thereby capturing most of the observed energy expenditure. A prior study noted a similar surplus of ATP production relative to biosynthetic requirements in *E. coli*^47^. Our combined observations suggest that substantial, yet underappreciated, ATP-consuming processes, such as protein chaperoning and macromolecule turnover, may be active during growth and contribute significantly to the overall cellular energy budget.

### Warburg Effect is required to support rapid growth rates

**Fig 3.**
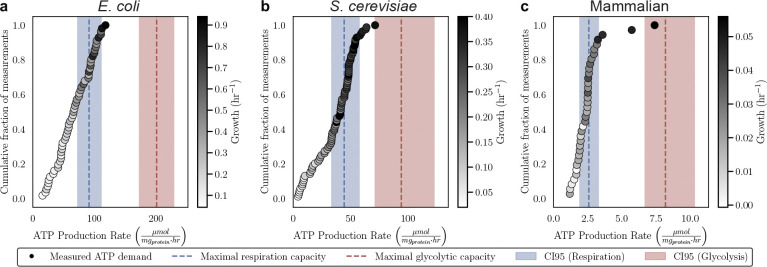
Glycolysis can support higher cellular ATP demands needed for fast growth. a-c, Empirical cumulative distribution functions (ECDFs) of measured ATP production rates (μmol mg protein^−1^ hr^−1^) for *E. coli* (a), *S. cerevisiae* (b), and mammalian cells (c). Each point is a ATP production rate measurement with point shading indicating the growth rate. Vertical dashed lines represent estimates of maximal respiration capacity (blue) and maximal glycolytic capacity (red), assuming complete allocation of the ATP-producing proteome to a single pathway. The corresponding shaded bands denote their 95% confidence intervals.

Previous studies have shown that glycolysis generates ATP at a higher rate per milligram of protein than respiration^48,16,17,8,14^. The metabolic switch enables rapidly proliferating cells to satisfy their increase ATP requirements efficiently by using the proteome allocated to ATP-generating enzymes. Although cells concurrently express proteins involved in both glycolysis and respiration, we estimated the maximal cellular ATP production rate under the hypothetical scenario in which all proteome allocated to ATP-generating enzymes was dedicated exclusively to either glycolysis or respiration. We generated empirical cumulative distribution functions (ECDFs) of measured ATP production rates for *E. coli*, *S. cerevisiae*, and mammalian cells (Fig. 3, points). Overlaid are the calculated maximal ATP production capacities via glycolysis (red dashed lines) and respiration (blue dashed lines), including their respective 95% confidence intervals (shaded areas) (Fig. 3). In these datasets, 26.8% of *E. coli*, 42.9% of *S. cerevisiae*, and 30.6% of mammalian cell measurements lie above the respiration line. This indicates that oxidative respiration alone is insufficient to meet the ATP demands required for growth in a substantial fraction of conditions. Based on our estimates, respiration alone can sustain maximum growth rates of 0.56 hr^−1^ for *E. coli*, 0.29 hr^−1^ for *S. cerevisiae*, and 0.024 hr^−1^ for mammalian cells. When compared to the fastest growth rates observed in each dataset, these values correspond to only 59.5%, 73.0%, and 43.0% of the maximum attainable rates, respectively. Importantly, none of the measured ATP production rates exceeded the estimated glycolytic capacity in any of the three organisms (Fig. 3, red line). Our analysis shows respiration alone appears insufficient to support the ATP production rate requirements of rapidly proliferating cells, whereas glycolysis (red) provide sufficient capacity under that same proteome allocation constraints (Fig 3). Our results align with the experimental demonstrations that glycolysis alone can meet the ATP demands of cells^49–51^. However, our results suggest that respiration becomes limiting in fast-growing cells due to its lower proteome efficiency. Based on the slope calculated in Figure 1C, we estimate that respiration alone cannot sustain cell proliferation at doubling times shorter than ~30 hours. We note that none of the cells could grow faster than maximal rate using glycolysis and most cells grew faster that maximal rate using respiration, suggesting that the Warburg Effect is required to achieve fast growth rates.

### Perturbation of ATP production yields corresponding changes in growth rate

We next investigated whether the linear relationship between ATP production rate and growth rate observed in Fig. 1 is causal by perturbing ATP production. To do so, we selectively inhibited each ATP-producing pathway, glycolysis or respiration, and measured corresponding changes in growth. Our aim was to determine whether the ATP production rate directly constrains proliferation. We selected three of the twelve previously studied cell lines, MCF7, U2OS, and C2C12, because they originate from distinct tissues, rely on different ratios of glycolysis and respiration for ATP production, and exhibit varying proliferation rates. If the linear relationship between ATP production rate and growth rate reflects a causal dependence, such that ATP production directly contributes to biomass production, then inhibition of ATP production should lead to reduced growth. Conversely, inhibition of growth should be accompanied by a corresponding reduction in ATP demand. Under this model, measurements from cells in which either growth or ATP production is impaired would be expected to align with the same linear trend, provided that the energetic cost of growth remains constant.

**Extended Data Fig. 3:**
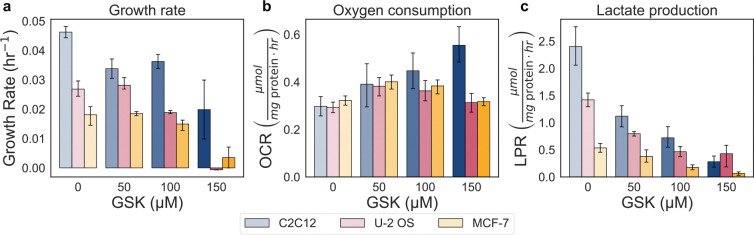
Effect of GSK-2837808A treatment on the growth, oxygen consumption, and lactate production rate. a-c, Dose–response to GSK (0, 50, 100, 150 μM) in MCF-7, U-2 OS, and C2C12 cells on the growth rate (μmol mg protein^−1^ hr^−1^), oxygen consumption rate (OCR) (μmol mg protein^−1^ hr^−1^), and lactate production rate (LPR) (μmol mg protein^−1^ hr^−1^). Error bars show 95% confidence intervals from 10,000 bootstrap resamples matched across measurements. Control reference points are shown as grey circles.

**Fig 4.**
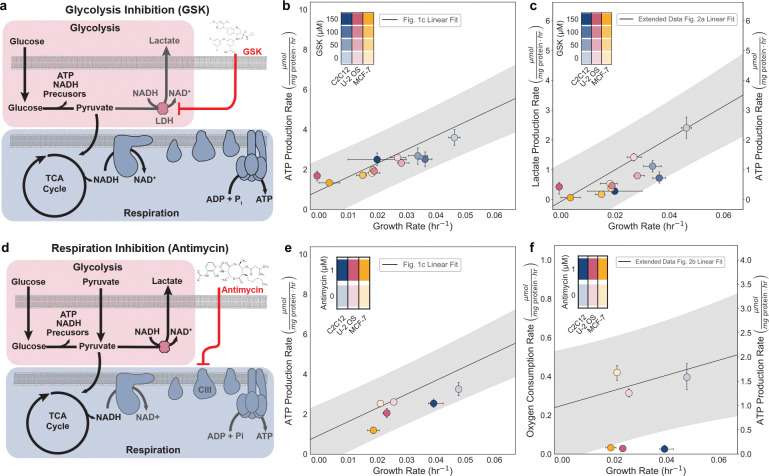
Glycolysis, not respiration, is needed to support high ATP flux during rapid growth. a, Schematic of LDH inhibition by GSK-2837808A (GSK), which prevents NAD^+^ regeneration and limits glycolytic ATP production. b-c, Growth–rate and total ATP production (μmol mg protein^−1^ hr^−1^) (b) or lactate production (μmol mg protein^−1^ hr^−1^) (c) dependence to GSK (0, 50, 100, 150 μM) in MCF-7, U-2 OS, and C2C12 cells. d, Schematic of respiratory inhibition by antimycin (complex III of the ETC). e-f, Growth–rate and total ATP production (μmol mg protein^−1^ hr^−1^) (e) or oxygen consumption rate (μmol mg protein^−1^ hr^−1^) (f) to antimycin (0 or 1 μM) in MCF-7, U-2 OS, and C2C12 cells. Linear fit and prediction band as indicated in Fig. 1c. Points represent group means; color denote cell lines and shading indicate inhibitor dose. Error bars show 95% confidence intervals from 10,000 bootstrap resamples matched across measurements.

To inhibit glycolysis, we targeted lactate dehydrogenase (LDH), the terminal enzyme in glycolysis. Inhibition of lactate dehydrogenase prevents the oxidation of NADH generated in glycolysis to NAD^+^, thus disallowing the pathway to be redox neutral and generate ATP independently (Fig 4a). We used a potent inhibitor of LDH, GSK-2837808A (GSK), which has an IC_50_ of 2.6 nM for human LDHA and 43 nM for human LDHB^52^ and has been shown to reduce lactate production in mammalian cells^53,54^. We treated cells with increasing concentrations of GSK (0, 50, 100, 150 μM). In all cell lines, we observed a dose-dependent decrease in both proliferation rate (hr^−1^) and lactate production rate, together with a decrease in total ATP production (Extended Data Fig 3). While the oxygen consumption rate displayed a modest upward trend, it was unable to compensate for the loss of glycolysis (Extended Data Fig 3). Additionally, we found that the growth rate and total ATP production rate (μmol μg^−1^ pathway protein min^−1^) declined in parallel (Fig. 4b), exhibiting a similar slope to Fig. 1c data. Our findings suggest a causal relationship between growth rate and ATP production rate.

**Extended Data Fig. 4:**
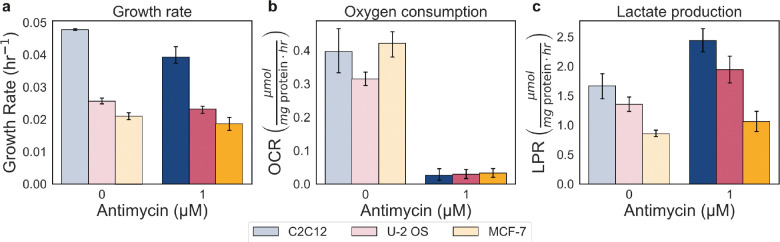
Effect of antimycin treatment on the growth, oxygen consumption, and lactate production rate. a-c, Dose–response to antimycin (0, 1 μM) in MCF-7, U-2 OS, and C2C12 cells on the growth rate (μmol mg protein^−1^ hr^−1^), oxygen consumption rate (OCR) (μmol mg protein^−1^ hr^−1^), and lactate production rate (LPR) (μmol mg protein^−1^ hr^−1^). Error bars show 95% confidence intervals from 10,000 bootstrap resamples matched across measurements. Control reference points are shown as grey circles.

To further test whether the relationship between ATP production and growth is causal, we inhibited a key component of mitochondrial respiration. Specifically, we inhibited cytochrome c reductase, also known as Complex III, in the electron transport chain using antimycin (Fig. 3d), acutely suppressing oxidative phosphorylation-derived ATP production. Even when ATP production from respiration abolished, we still observe a linear relationship between growth and ATP production, reinforcing our hypothesis that growth rate is causally linked to the cellular ATP production rate (Fig. 3e). We observed that the oxygen consumption falls outside the 95% confidence interval of the prediction (Fig 4f). Here, the growth rates remain high because increased glycolytic rate compensates for the loss of ATP from oxidative phosphorylation (Extended Data Fig. 4). Minimal effect on growth has been reported with other ETC inhibitors, including antimycin for various mammalian cell lines^50,55,56^.

Our results provide quantification of how glycolysis and oxidative phosphorylation compensate for one another to sustain ATP production and maintain proliferation rates under pathway-specific inhibition. We observed a causal relationship between the ATP production rate and growth, such that inhibiting the ATP production led to a linear decrease in growth. Furthermore, we demonstrate that glycolysis plays a predominant compensatory role in supporting ATP production, when oxidative phosphorylation is impaired. Oxidative phosphorylation partially compensates for reduced glycolysis, but its capacity is insufficient to fully sustain growth in faster-growing cells reliant on glycolysis. Together, these data suggest that glycolysis is needed, but oxidative phosphorylation is not strictly required, to sustain rapid growth.

### Perturbation of growth rate yields corresponding changes in ATP production

**Extended Data Fig. 5:**
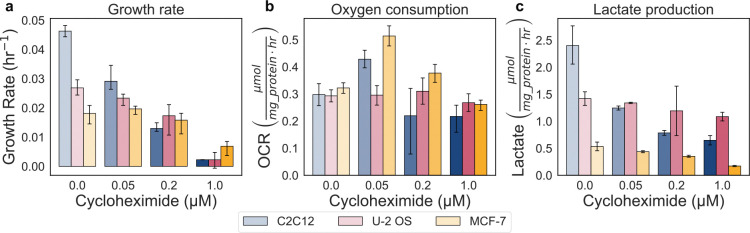
Effect of cycloheximide treatment on the growth, oxygen consumption, and lactate production rate. a-c, Dose–response to cycloheximide (0, 0.05, 0.2, 1 μM) in MCF-7, U-2 OS, and C2C12 cells on the growth rate (μmol mg protein^−1^ hr^−1^), oxygen consumption rate (OCR) (μmol mg protein^−1^ hr^−1^), and lactate production rate (LPR) (μmol mg protein^−1^ hr^−1^). Error bars show 95% confidence intervals from 10,000 bootstrap resamples matched across measurements. Control reference points are shown as grey circles.

**Fig 5.**
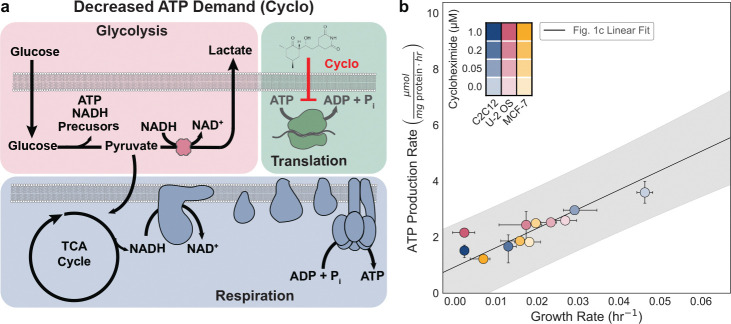
Translation inhibition lowers growth and cellular ATP demand. a, Schematic of cycloheximide (Cyclo) activity, which blocks protein translation and lowers both growth rate and cellular ATP demand. b, Growth–rate and total ATP production (μmol mg protein^−1^ hr^−1^) dependence to cycloheximide treatment (0, 0.05, 0.2, 1 μM) in MCF-7, U-2 OS, and C2C12 cells. Linear fit and prediction band as indicated in Fig. 1c. Points represent group means; color denote cell lines and shading indicate inhibitor dose. Error bars show 95% confidence intervals from 10,000 bootstrap resamples matched across measurements.

We asked whether reducing growth by inhibiting translation produces proportional decreases in ATP production. Protein synthesis is one of the most energetically expensive cellular processes, consuming four ATP equivalents per peptide bond. Cycloheximide is a potent inhibitor of eukaryotic translation that binds the 60S ribosomal subunit and blocks elongation (Fig 5a). We observed a dose-dependent decrease in growth rate across MCF-7, U-2 OS, and C2C12 cells with increasing cycloheximide treatment (0, 0.05, 0.2, 1 μM) (Fig. 5b, Extended Data Fig. 5)^57^. The decline in growth was accompanied by linear reductions in ATP, demonstrating that reducing the growth rate yields a causal reduction of the ATP production rate (Fig 5b). Interestingly, we also observed growth dependent decrease in the lactate production rate, whereas oxygen consumption declined only at the highest dose (Extended Data Fig. 5). A similar preferential decrease in the glycolytic rate was previously reported in *S. cerevisiae*, where cycloheximide treatment reduced ethanol production^8^, and in *E. coli*, where chloramphenicol-mediated translation inhibition reduced acetate production^47^. Together, we found that cells adjust their ATP production rate as the ATP demand decreases through modulation of the growth rate. This finding, coupled with results from Fig. 4, support a bidirectional causality where ATP production rates influence growth, and conversely, growth rates significantly dictate ATP production requirements. Our data support the conclusion that the majority of ATP production at high growth rates are predominantly associated with new biomass synthesis rather than cellular maintenance.

### Increased ATP demand limits cell growth

**Extended Data Fig. 6:**
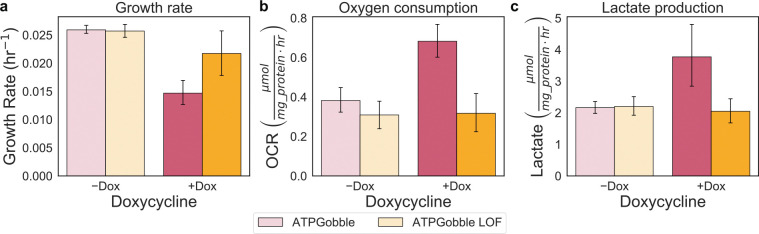
Effect of ATPGobble expression on the growth, oxygen consumption, and lactate production rate. a-c, Response to ATPGobble or ATPGobble Loss of Function (LOF) in U-2 OS cells on the growth rate (μmol mg protein^−1^ hr^−1^), oxygen consumption rate (OCR) (μmol mg protein^−1^ hr^−1^), and lactate production rate (LPR) (μmol mg protein^−1^ hr^−1^). Error bars show 95% confidence intervals from 10,000 bootstrap resamples matched across measurements. Control reference points are shown as grey circles.

**Fig 6.**
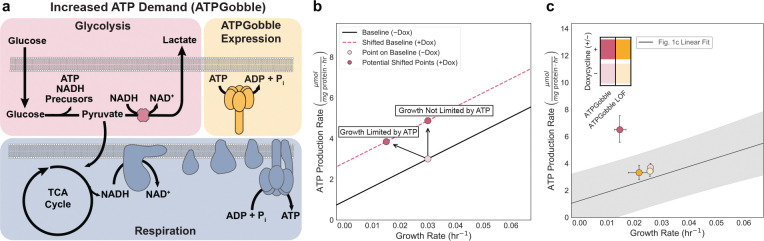
Increasing ATP demand with ATPGobble reduces growth. a, Schematic of ATPGobble activity, which increases cellular ATP demand. b, Theoretical shift of ATPGobble (+Dox) points relative to (−Dox), illustrating the potential outcomes where ATP production constrains or does not constrain the cellular growth rate. c, Growth–rate and total ATP production (μmol mg protein^−1^ hr^−1^) dependence to ATPGobble expression U-2 OS cells. Linear fit and prediction band as indicated in Fig. 1c. Points represent group means; color denote ATPGobble cell lines and shading indicate inhibitor dose. Error bars show 95% confidence intervals from 10,000 bootstrap resamples matched across measurements.

Our results so far suggest that ATP production scales with growth and that inhibiting either ATP supply or biomass synthesis correspondingly impairs both processes. To further test whether ATP production capacity is limiting for growth, we sought an orthogonal approach where we increased the ATP demand without changing biosynthetic needs. To this end, we expressed a recently developed genetically encoded metabolic tool (GEMM), termed ATPGobble, which selectively increases ATP hydrolysis without affecting cell signaling or biomass production (Fig 6a). ATP Gobble is a modified F_1_ ATPase lacking the peripheral stalk subunit, causing it to rotate in reverse and hydrolyze ATP into ADP, thereby creating an additional ATP sink^58^. The expression of ATPGobble is akin to increasing the maintenance ATP demand, where we would expect an upward translation of the line, while the relationship between the ATP production rate and growth would be maintained (Fig 6b). If ATP production is not limiting for proliferation, we would expect the cell to tolerate this added energy burden without compromising its growth rate (Fig. 6b). However, if the cell’s capacity to generate ATP sets a ceiling on its growth potential, increased ATP consumption should reduce proliferation, even if the cell compensates by upregulating ATP production (Fig 6b). As expected, induction of ATPGobble in U-2 OS cells (+DOX) increased the total ATP production rate with a marked rise in lactate production and oxygen consumption relative to −DOX and LOF controls (Fig. 6c, Extended Data Fig. 6). Importantly, we observed slowed growth by ~2-fold relative to uninduced and LOF controls (Fig. 6c, Extended Data Fig. 6). Therefore, the ATP production rate compensation was insufficient to maintain the original proliferation rate. Our results provide direct evidence that ATP production capacity can constrain growth in mammalian cells as has been demonstrated in microbes^23^.

## Discussion

Our study reveals a quantitative relationship between ATP production and cellular growth in mammalian cells, which is conserved across evolutionarily distant organisms. The similarity in slopes across bacteria, yeast, and mammalian cells indicates a shared energetic cost of producing one unit of biomass. These empirical slopes are closely approximated by theoretical ATP demands for synthesizing proteins, lipids, and nucleotides, which capture about ~85% of total ATP expenditure. While many previous studies have noted the linear relationship between ATP and growth in microbes, we are aware of only a single study using one cell line for mammalian cells. Kilburn et al. 1969 determined a linear relationship between ATP production and growth rate for a single mouse cell line, in which variable growth rates were obtained by adjusting pO_2_. Though the study concluded that a linear relationship between the ATP production rate and growth, we saw one major issue with their calculations in that the study assumes a yield of 38 ATP per molecule of glucose through respiration. In our analysis, we have revisited recent studies examining the H^+^/ATP ratio of ATP synthase and proton leak in mammalian cells^59^. Based on these assessments, we propose a revised ATP yield from respiration, suggesting a yield of 24 ATP molecules per glucose molecule. Therefore, we both find a linear relationship between ATP production and growth through different approaches, we out forth a different slope based on a different ATP yield per molecule of glucose from respiration.

A key reason microbes achieve faster growth rates than mammalian cells is their ability to generate substantially more ATP per unit biomass. As shown in our previous study^14^, microbes exhibit much higher specific activities for ATP production than mammalian cells, meaning each gram of pathway protein can turn over substrate and produce ATP at a faster rate. Combined with larger proteome fractions allocated to energy metabolism, this allows microbes to sustain ATP fluxes that are an order of magnitude higher per unit biomass. Since the ATP cost of biosynthesis per gram of new cell mass is broadly conserved across species, the limiting factor is not ATP demand but supply. Thus, microbial cells can divide faster than mammalian cells because they can marshal proportionally greater ATP production capacity relative to their biomass.

The ATP production rate adjusts to meet the cellular ATP demand during increased growth, shifting the production strategy from respiration to glycolysis, known as the Warburg Effect^16,39,60^. Previous work has demonstrated that the Warburg Effect is the result of faster, more proteome efficient method of ATP production and necessary for meeting high ATP demands^48,61,62,16,17,14^. Here we argued that the high ATP demands of fast growth could not be satisfied by respiration-alone in *E. coli*, *S. cerevisiae*, and mammalian cells, even if the entire ATP-producing proteome were populated with proteins from this pathway (Fig. 3) and therefore glycolysis-mediated ATP production is necessary for achieving fast growth rates. Using small molecular inhibitors of either glycolysis or respiration, we directly tested the premise and found that respiration alone was insufficient to meet ATP demands at high growth rates (Fig. 4). Our findings align with a previous study in which double knockout of LDHA and LDHB in human colon adenocarcinoma (LS174T) and murine melanoma (B16-F10) cells led to a two-fold reduction in growth rates compared to wild-type cells^63^. Conversely, glycolysis-alone was able is compensate for the loss of respiration, sustaining growth at nearly unchanged rates (Fig. 4), corroborated by previous studies has been lines^50,55,56^.

Inhibition of protein synthesis with cycloheximide resulted in a dose-dependent decrease in both growth rate and ATP production (Fig. 5). The decline in ATP production was primarily attributed to a loss of glycolytic activity, consistent with prior observations in *S. cerevisiae*^8^. In previous work, we reanalyzed proteomic data from *S. cerevisiae* subjected to increasing doses of cycloheximide and observed a progressive reduction in the proteome fraction allocated to ATP production^14^. This shift was accompanied by downregulation of glycolytic rates and relatively sustained levels of respiratory rates, aligning with their distinct ATP production efficiencies. We hypothesize that a similar proteomic reallocation underlies the metabolic response in mammalian cells, whereby glycolysis is selectively downregulated while respiration is maintained. Remarkably, the linear relationship between ATP production and growth was preserved, indicating that cells downregulate ATP production in proportion to reduced biosynthetic demand. These findings support a bidirectional link between growth and ATP production and reinforce the conclusion that the majority of ATP demand at high growth rates is driven by biomass synthesis rather than maintenance.

Finally, we observed impaired growth upon enforced upregulation of ATP consumption using the ATP Gobble construct, providing direct evidence that the capacity for cellular energy production can constrain proliferative potential. This finding is particularly notable considering longstanding debate within the cancer metabolism field regarding whether ATP supply poses a limiting barrier to cell growth^10,25,64^. Previous results demonstrated the expression of ATPGobble in hTERT-RPE1 cells lowered the ATP/ADP ratio, activated AMPK, and induced a two-fold increase in both glycolytic and respiratory fluxes while exhibiting slowed or stopped proliferation^58^. In future studies it would be impactful to see if ATP production limitations exist in proliferating cancer cells *in vivo*, especially given that one study observed a lower ATP production rate in tumors as compared to adjacent, nonproliferating tissues of origin^11^. Collectively, these findings position energy metabolism as a fundamental constraint on growth, with implications for understanding both cellular physiology and the metabolic reprogramming observed in cancer and other proliferative states.

## Methods

### Cell lines and cell culture

All cell lines were culture in Dulbecco’s modified eagle’s medium [DMEM (Gibco^™^ 12800082), 3.7 g/L NaHCO_3_, 10% FBS (Gibco^™^ 10437028), and 100 U / mL Penicillin-Streptomycin (Gibco^™^ 15140122)]. All experiments were performed in the absence of Penicillin-Streptomycin. The following cells lines were used and obtained from the University of California, Berkeley Cell Culture Facility: C2C12 (ATCC CRL-1772), HeLa (ATCC CCL-2), U-2 OS (ATCC HTB-96), A549 (ATCC CCL-185), SK-BR-3 (ATCC HTB-30), NCI-H1703 (ATCC CRL-5889), PC-3 (ATCC CRL-1435), A431 (ATCC CRL-1555), and MCF-7 (ATCC HTB-22), B16-F10 (ATCC CRL-6475). Huh-7^65^ and MC38^66^ cells were kindly provided Anders Näär’s and Michael DuPage’s labs, respectively.

### Cell Proliferation

Cell proliferation was quantified using nuclei counting and image analysis on a Cytation 1 imaging reader with Gen5 3.05 software. Cells were seeded into black, clear-bottom 96-well plates (Costar 3904) at densities optimized for each line, 300–500 cells per well, in 200 μl complete medium. Plates were harvested daily for fixation and staining for up to 7 days. Cells were fixed in 4% paraformaldehyde (PFA, diluted from 16% stock in PBS, filtered through 0.22 μm membrane) for 15 min at 37 °C. Wells were then washed and stained with Hoechst nuclear dye (final concentration 1 μg/ml, freshly prepared from 10,000× stock in PBS). Plates were sealed with light-blocking film and stored at 4 °C until imaging. Imaging was performed using the Cytation 1 system with excitation and emission settings optimized for Hoechst and images were processed using Gen5 software. Automated segmentation parameters (object size, rolling ball diameter, and thresholding) were adjusted as needed to optimize nuclear identification. Proliferation rates were quantified as the number of nuclei per well. For each plate, outer wells were removed to growth edge effects. To ensure accurate growth-rate estimation, wells with improperly low nuclei counts (<100 cells) were excluded from analysis. Cell proliferation was modeled as exponential growth (Equation 1), and growth rates were calculated by linear regression of the natural logarithm of cell number versus time:

[1] $$ln\left(N_{t}\right)=ln\left(N_{0}\right)+\mu \cdot t$$

where $N_{0}$ and $N_{t}$ are the numbers of nuclei counted at the initial time (0) and at time t, respectively, t is the time elapsed (days), and μ is the exponential growth rate constant determined from the regression slope. Time points after cultures reached saturation were excluded from analysis, as growth was no longer exponential once wells became confluent. Rates were converted to per-hour units (hr^−1^) by dividing μ by 24. Growth metrics were reported per treatment group using replicate-level regression fits rather than fitting to averaged growth curves.

### Production of cells stably expressing ATPGobble under control of doxycycline promoter

Doxycycline-inducible pLVX-TetOne vectors encoding codon-optimized *E. coli* F1-ATPase subunits atpA, atpG, and atpD (Thermo Fisher codon optimizer; backbone: pLVX-TetOne-Puro, Takara #631849) were used for ATPGobble. For atpA and atpD, the puromycin cassette was replaced with blasticidin (Addgene #183751) and zeocin (Addgene #161748), respectively; a catalytically inactive control carried an atpD(K155Q) mutation generated by site-directed mutagenesis (NEB E0554S). Lentivirus was produced using HEK293T cells. Briefly, 5 × 10^5^ HEK293T cells were seeded per well of a 6-well plate in 2 ml high-glucose DMEM (Life Technologies, 11995) supplemented with 10% FBS. After 24 hr, the medium was replaced with fresh DMEM, and cells were transfected with a DNA mixture containing 500 ng psPAX2 (Addgene plasmid #12260), 50 ng pMD2.G (Addgene plasmid #12259), and 500 ng of the lentiviral vector of interest. Plasmids were combined in Opti-MEM (Life Technologies, 31985–070) to a final volume of 50 μl. In parallel, 3 μl X-tremeGENE 9 reagent (Roche, 06365787001) was diluted in 50 μl Opti-MEM. The DNA solution was added dropwise to the reagent solution, incubated at room temperature for 30 min, and then added to cells. Two days after transfection, virus-containing supernatant was harvested, centrifuged at 500 × g for 5 min to remove cell debris, and filtered through a 0.45 μm PES filter (Millipore Millex-HP, SLHP033RS). Viral supernatant was used immediately for infection of target cells. Target cells were seeded the day before infection at densities appropriate for each line with 50,000 cells/well in 2 ml of growth medium per well of a 6-well plate. After 24 hr, polybrene was added (final concentration 8 μg/ml), and cells were exposed to 1000 μl of viral supernatant of each construct. Three wells were left uninfected to serve as a control for each antibiotic selection. Medium was replaced 24 hours post-infection. At 48 hours post-infection, selection was initiated by adding blasticidin (5 μg/mL), zeocin (100 ng/mL), puromycin (10 mg/mL), and geneticin (500 μg/mL). Infected cultures were maintained under antibiotic pressure until all control cells were eliminated.

### Measurement of cell volume

During each cell passage, the Coulter Z2 Counter Cell Particle Analyzer (Beckman) was used to determine the concentration and volume of cells. The average volume (fL) and standard deviation of each cell line are reported and used throughout to convert cell count to mg protein.

### Cell Protein Concentration Measurement

Protein concentration was determined using bicinchoninic acid (BCA) assay. One million cells were collected in a 2-mL centrifuge tube. Cells were pelleted at 600 g. Media was aspirated. Cells were washed with phosphate-buffered saline, spun, and aspirated twice to remove protein contained in the media. Cells were resuspending in 500 μL of lysis buffer containing 1% sodium dodecyl sulfate and incubated at 90°C for 10 min. Samples were prepared for analysis in triplicate as instructed in the Pierce^™^ BCA Protein Assay Reagent Kit (Thermo Fisher Scientific 23225). Samples intensity was measured at 562 nm using BioTek Cytation1. Protein concentrations per cell volume for each analyzed mammalian cell line as compared to a bovine serum albumin standard.

### Calculation of cellular ATP production rates from glycolysis and respiration rates

ATP yields for glycolysis and respiration were assigned based on experimentally derived values and theoretical stoichiometric calculations that account for ATP produced in glycolysis, the TCA cycle, and oxidative phosphorylation. As described previously, fermentative glycolysis yields 2 ATP per glucose in *E. coli*, *S. cerevisiae*, and mammalian cells^14^. Respiration yields were determined by quantifying NADH and FADH_2_ production, the number of protons translocated per electron through each organism’s electron transport chain, proton leak, and the proton cost of ATP synthesis and , as detailed in our previous study^14^. Resulting effective ATP yields per glucose were 20 ATP for *E. coli*, 16 ATP for *S. cerevisiae*, and 24 ATP for mammalian cells^14^. In *E. coli*, Pta–AckA pathway is the major route of acetate production from glucose. This pathway generates 4 NADH that are oxidized through the electron transport chain, yielding approximately 10 ATP per molecule of glucose^14^. These organism-specific yields were used to stoichiometrically convert glycolytic byproducts production or oxygen consumption rates to ATP production rates.

### Calculation of maximal cellular ATP production rate from glycolysis and respiration

To estimate the maximal ATP production rate $V_{ATP}$ achievable through glycolytic or respiratory pathways that could be supported by the total ATP-producing proteome $\left(\phi_{\mathrm{total}}^{ATP}\right)$ in the extreme case in which it is only occupied by enzymes for a single pathway, we combined three previously derived parameters^14^:

1. The maximal glucose uptake rate for each pathway $\left(V_{\max}^{\mathrm{pathway}}\right)$,
2. The ATP yield per glucose ($\gamma_{\mathrm{pathway}}$), and
3. The fraction of total proteome allocated to ATP-producing enzymes $\left(\phi_{\mathrm{total}}^{ATP}\right)$.

The overall rate of ATP production was calculated as:

[2] $$V_{ATP}=V_{max}^{\mathrm{pathway}}\cdot \gamma_{\mathrm{pathway}}\cdot \phi_{\mathrm{total}}^{ATP}$$

where $V_{ATP}$ is expressed in μmol ATP per mg protein^−1^ per min^−1^.

To account for experimental variability from the datasets in enzyme abundance, pathway allocation, and substrate uptake rates, a nonparametric bootstrap resampling procedure (10,000 iterations) was implemented. For each iteration, glucose uptake rates for fermentation and respiration as well as total proteome allocations to ATP-producing enzymes were randomly sampled with replacement from their respective empirical distributions. For each bootstrap iteration, the maximal rate of ATP production was calculated for glycolysis or respiration (Equation 2), where $\gamma_{\mathrm{glycolysis}}$ and $\gamma_{\mathrm{respiraation}}$ were pathway-specific ATP yields as described in *Calculation of cellular ATP production rates from glycolysis and respiration rates.* After 10,000 bootstrap iterations, the resulting distributions of $V_{ATP}$ values were sorted to determine the 2.5th and 97.5th percentiles, providing 95% confidence intervals for the maximal ATP production achievable via glycolysis or respiration occupancy for the whole ATP-producing proteome in each organism.

### Measurement of lactate production rate

Lactate production rates (LPR) of cell lines were measured using the L-Lactate Assay Kit-I (Colorimetric) (Eton Bioscience 120001400A). Cells were seeded at the volumetric equivalent of 0.5 million HeLa cells, where the average HeLa cell volume was measured to be 2300 fL, per well of a 6-well plate in 3 mL of DMEM and were incubated at 37°C in 5% CO2 incubator. The medium was replaced 24 hours later with 3 mL of the assay medium [DMEM (Gibco^™^ 12100061), 10 % dialyzed FBS (Gibco^™^ 26400044) and 3.7 g/L NaHCO_3_]. Two hundred μL of media was collected every hour for 4 hours and immediately frozen on dry ice. Samples were stored at −80°C until analyzed. At the end of the 4 hour sampling period, cells were trypsinized and counted using the coulter counter. To analyze the samples, 50 μL of sample was combined with 50 μL L-Lactate assay solution. The absorbance at 490 nm was measured for 45 min at 37°C on the BioTek Cytation1. The slope of each well was determined. A standard curve was made by plotting the slope of OD490 nm values for each L-Lactate standards as a function of L-Lactate concentration. The L-Lactate concentration of each biological sample was determined using the equation obtained from the linear regression of the standard curve. L-Lactate in each sample was converted from concentration to moles per g protein using obtained cell counts and averaged protein concentrations per cell volume for each mammalian cell line. Linear regression was performed across the four-hour time course to determine the lactate production rate for each cell line.

### Measurement of oxygen consumption rate

Oxygen consumption rates (OCR) of cell lines were measured with the Agilent Seahorse XFe24 Analyzer. Cells were seeded at the volumetric equivalent of 100,000 HeLa cells, where the average HeLa cell volume was measured to be 2300 fL, per well of XFe24 cell culture microplates in 150 μL of DMEM and were incubated at 37°C in 5% CO2 incubator. The medium was replaced 24 hours later with 500 μL of the assay medium [DMEM (Gibco^™^ 12100061), 10 % dialyzed FBS (Gibco^™^ 10437028) and 5 mM HEPES-KOH, pH 7.4] and the plates were placed in the Agilent Seahorse XFe24 Analyzer for OCR measurements. Each measurement was performed over 4 min after a 2 min mix and 2 min wait period. Basal measurements were collected 3 times, 3 measurements were collected after injection of oligomycin (final concentration of 1 μM), 3 measurements were collected addition of an initial injection FCCP (final concentration of 3 μM), 3 measurements were collected after a subsequent addition of FCCP (final concentration of 6 μM), 3 measurements were collected after addition of antimycin A and rotenone (final concentration of 1 μM each). Each drug was injected as a concentrated 50 μL solution of the assay medium. Any negative OCR values were reported as zero^67–69^. the end of the measurements, cells were trypsinized and counted using the Coulter Z2 Counter Cell Particle Analyzer (Beckman). Oxygen consumption rates were converted to μmol per mg protein per min using obtained cell counts and averaged protein concentrations per cell volume for each mammalian cell line.

### ATP Production Rate Estimates for Macromolecular Biosynthesis

To quantify the ATP required to support cellular biomass synthesis during proliferation, we estimated the contribution of each major macromolecular class, protein, RNA, DNA, and lipid, to the total biosynthetic ATP burden. For each organism, the ATP demand was determined by multiplying (i) the cellular mass fraction of each macromolecule by (ii) the ATP required to synthesize one milligram of that component (Equation 3):

[3] $$ATP\;demand\;=\sum_{i}\;\left(mass\;fraction\;\left.\cdot ATP{\;cost}_{i}\right)\right.$$

where i∈{protein,RNA,DNA,lipid}.

The fractional composition of cellular biomass was obtained from literature sources for *E. coli*, *S. cerevisiae*, and mammalian cells. Average biomass compositions by dry weight was subdivided into major macromolecular classes: proteins, lipids, nucleic acids (DNA and RNA) for *E. coli*^3,70,71^, *S. cerevisiae*^72–74^; mammalian cells^75–77^.

The ATP costs (μmol ATP per mg macromolecule) for biosynthesis were calculated for each category. Protein synthesis includes ATP for amino acid activation and GTP for ribosomal elongation. The total energetic cost is approximately 4 ATP equivalents per amino acid (1 ATP for tRNA charging, 2 GTP for elongation, initiation/termination overhead)^3,78,79^. We assumed an average amino acid to have a mass of 110 Da^6^. Fatty acid synthesis cost was estimated by summing the ATP required for acetyl-CoA carboxylation (7 ATP per C16 chain) and the reducing power required for fatty acid elongation (14 NADPH per C16 chain × 2.5 ATP equivalents per NADPH^80^), yielding a total of 42 ATP equivalents per fatty acid^81^. We assumed all lipids be palmitic acid with a mass of 256 Da^6^. *De novo* purine synthesis requires 12 ATP per nucleotide; pyrimidine synthesis requires 8 ATP^82^. Assuming a 1:1 purine:pyrimidine ratio, the average ATP cost is 10 ATP per nucleotide at 330 Da per nucleotide.

## Extended Data

## Data Availability

All data are available in the main text or the supplementary materials.
